# Supplementary material for: Atomic motifs govern the decoration of grain boundaries by interstitial solutes
Source: Nat Commun. 2023 Jun 15;14:3535. doi: 10.1038/s41467-023-39302-x (PMC10267137; doi:10.1038/s41467-023-39302-x)
Supplement: Supplementary file 1 — Supplementary information [file 41467_2023_39302_MOESM1_ESM.pdf]

Supplementary Information for

Atomic motifs govern the decoration of grain boundaries by interstitial solutes

Xuyang Zhou[1,2,*], Ali Ahmadian[2], Baptiste Gault[1,3], Colin Ophus[4], Christian H. Liebscher[2], Gerhard Dehm[2], Dierk Raabe[1,*]

[1] Department of Microstructure Physics & Alloy Design, Max-Planck-Institut für Eisenforschung GmbH, 40237 Düsseldorf, Germany.

[2] Department of Structure & Nano- / Micromechanics of Materials, Max-Planck-Institut für Eisenforschung GmbH, 40237 Düsseldorf, Germany.

[3] Department of Materials, Royal School of Mines, Imperial College London, SW7 2AZ London, UK.

[4] National Center for Electron Microscopy, Molecular Foundry, Lawrence Berkeley National Laboratory, Berkeley, California 94720, USA

[*]Corresponding to: x.zhou@mpie.de; d.raabe@mpie.de

This PDF file includes:

Supplementary Fig. 1 to Fig. 13

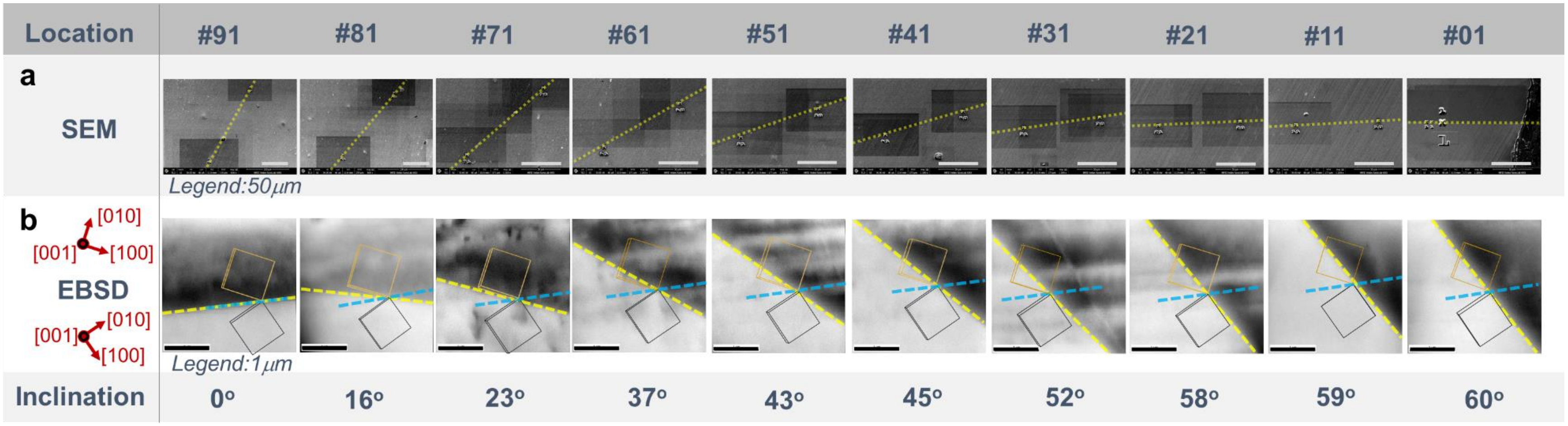

Supplementary Fig. 1 | Scanning electron microscope (SEM) and EBSD characterization of the Fe bicrystal. The [001] tilt Σ5 GBs have been obtained by the Bridgeman method. The examined GBs have a total length of approximately 1 cm, with the different sections labeled with numbers from #01 to #91. **a** shows the SEM images with the dashed yellow lines superimposed to highlight the GBs. Note that the sample was fixed without rotation during the SEM imaging. The orientation of the GB changed monotonically from one side of the sample to the other. **b** contains the EBSD scans of the sections from #01 to #91. Because the SEM and EBSD images were acquired with different equipment, there is a fixed rotation (approximately 70°) between these two sets of images. In each EBSD sub image, the dashed yellow line indicates the location of the GB. The orange and grey squares mark the orientations of the top and bottom, respectively. The blue dashed line was drawn as the inner angle bisector between the [100] direction of the upper grain and the [010] direction of the lower grain. There are two angle bisectors when considering the translation of the unit cells. Here we consistently use the representation shown in b. With that, the inclination, i.e. the angle between the GB plane and the inner bisector, is listed under each EBSD image.

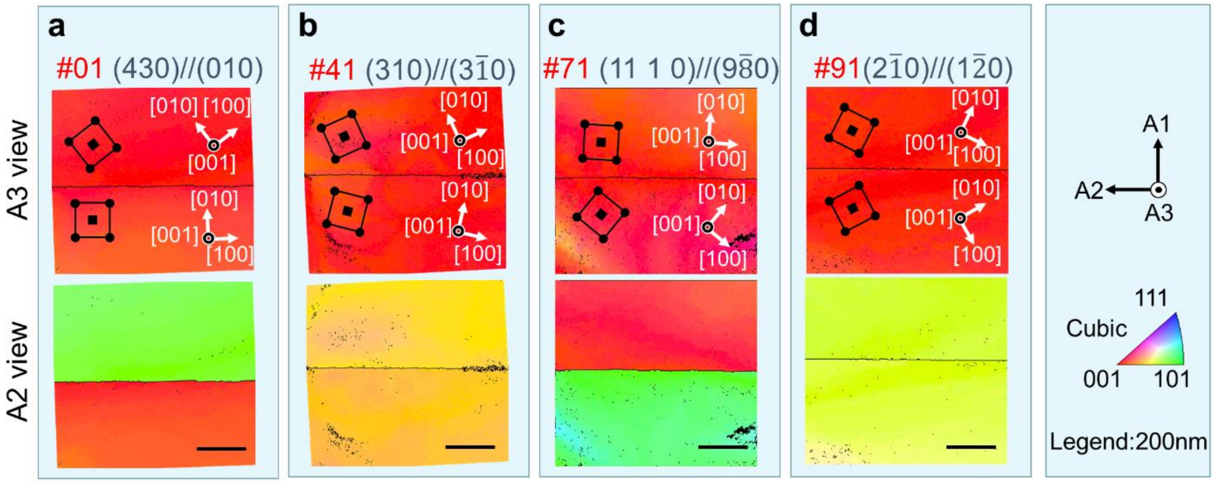

Supplementary Fig. 2 | Nanoscale orientation mapping of Σ5 GBs for selected regions. **a-d** are the reconstructions for GBs #01 (430) // (010), #41 (310) // (3$\bar{1}$0), #71 (11 1 0) // (9$\bar{8}$0), and #91 (2$\bar{1}$0) // (1$\bar{2}$0) from the precession assistant 4DSTEM data sets, to show the A3 view (top images, sample coordinates A1-A3 were defined in the last column of the figure) and the A2 view (bottom images) of the orientation of the grains. The superimposed squares mark the orientations of the grains. The coordination systems for the upper and lower grains are also included in the orientation maps of the A3 view.

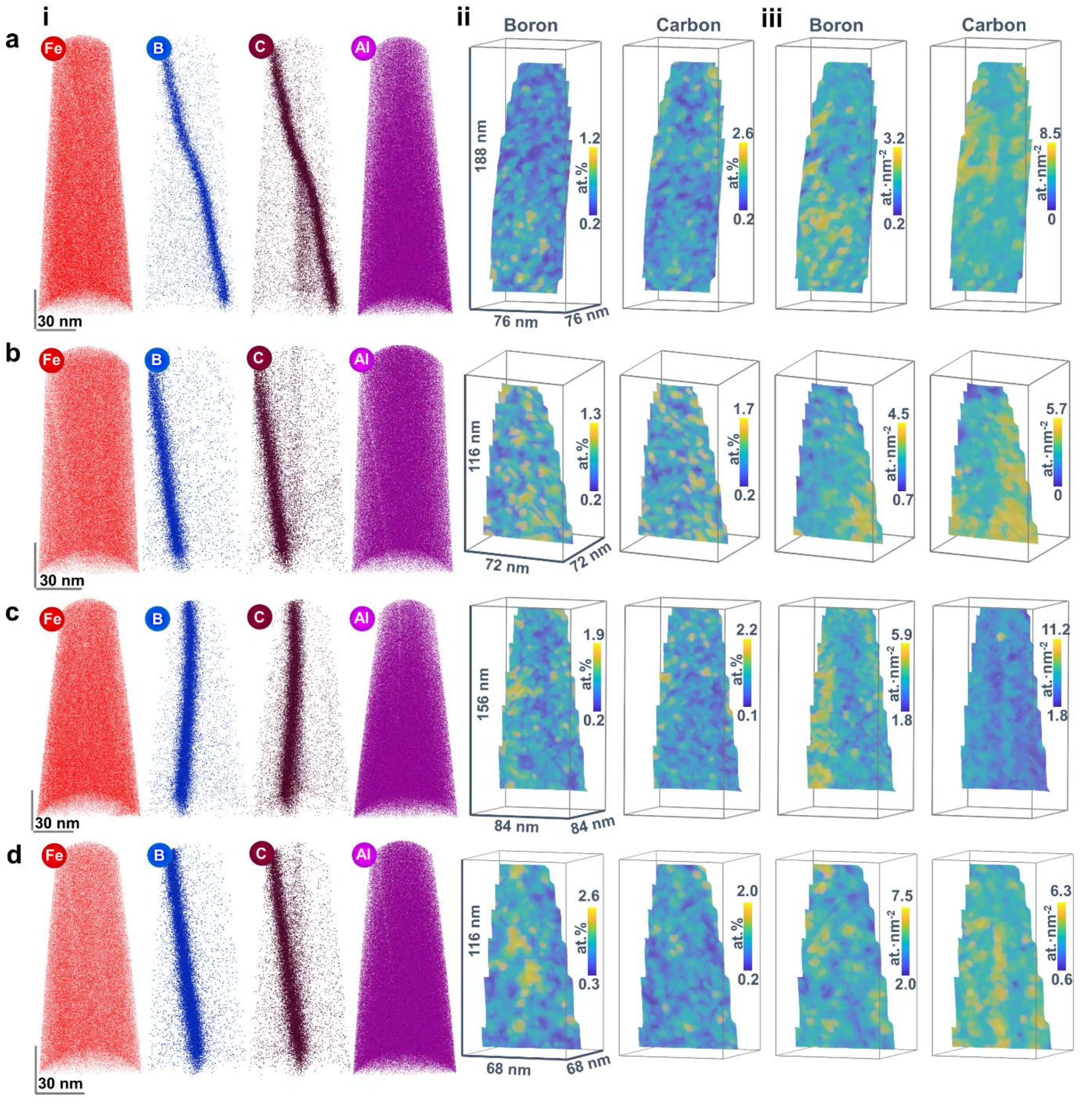

Supplementary Fig. 3 | Local chemical composition analysis of Σ5 GBs for selected regions. **a-d** are the APT analyses for GBs #01 (430) // (010), #41 (310) // (3$\bar{1}$0), #71 (11 1 0) // (9$\bar{8}$0), and #91 (2$\bar{1}$0) // (1$\bar{2}$0), respectively. In each row, **i** shows, from left to right, the atom maps of Fe (only show 3 at. % of total Fe atoms), B, C, and Al; **ii** the composition map and **iii** the interfacial excess map of solute B (left) and C (right) for the GBs found in the APT sample shown in i.

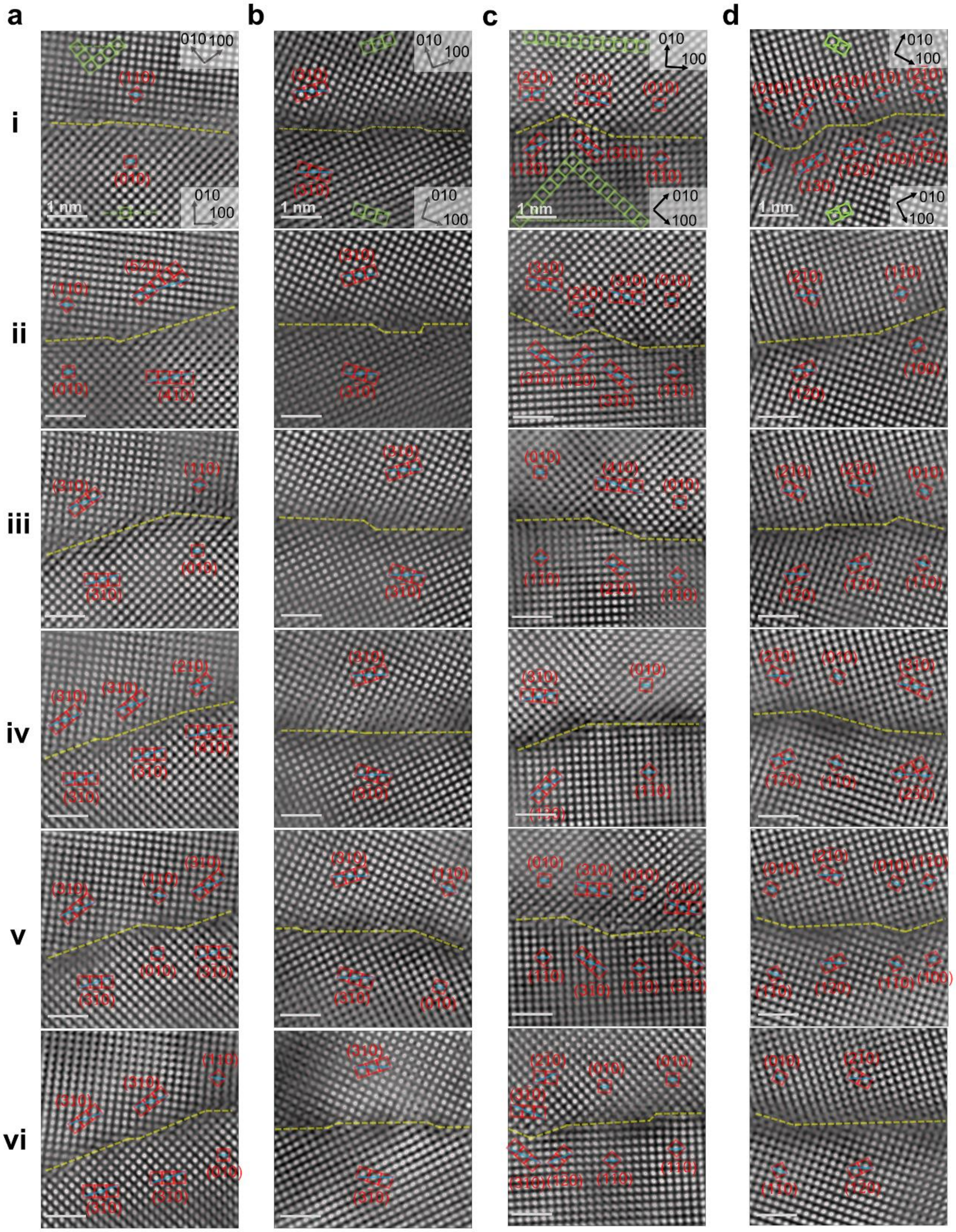

Supplementary Fig. 4 | Analysis of local facet structure of $\Sigma5$ GBs for selected regions. a-d are high resolution HAADF-STEM images of $\Sigma5$ GBs with mesoscopic GB planes of #01 (430) // (010), #41 (310) // (3$\bar{1}$0), #71 (11 1 0) // (9$\bar{8}$0), and #91 (2$\bar{1}$0) // (1$\bar{2}$0), respectively. The yellow lines indicate the positions of the GBs. The red squares and blue lines help identify the local GB planes. The green squares and lines help identify the mesoscopic GB planes.

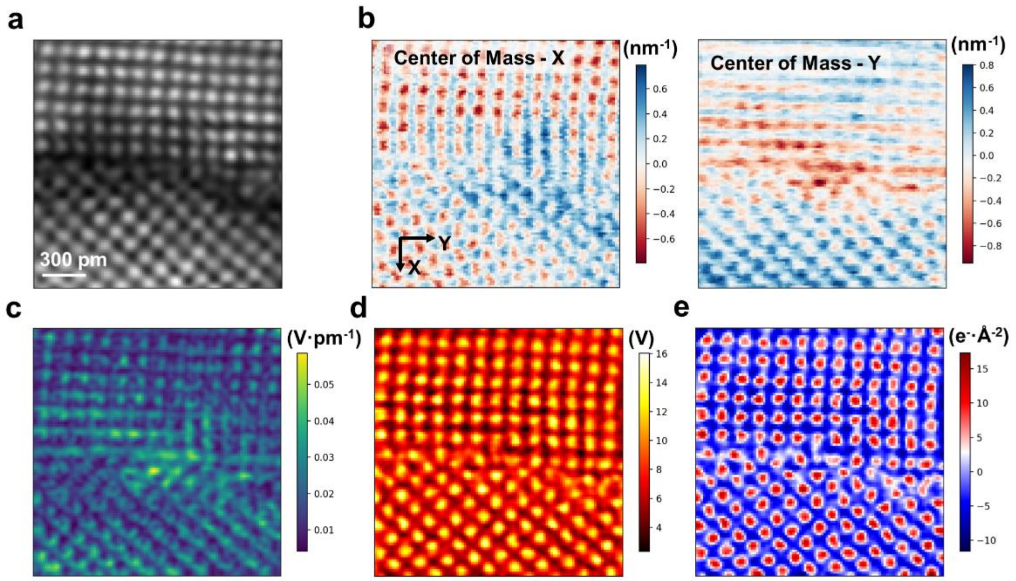

Supplementary Fig. 5 | Experimental DPC-4DSTEM reconstruction for the (110) // (010) atomic motif. a Reconstructed dark-field image. **b** Change of the center of mass (c.m.) of the transmitted beam in X (left image) and Y (right image) directions. **c** Electric field magnitude. **d** (Projected) electrostatic potential. **e** Charge-density map.

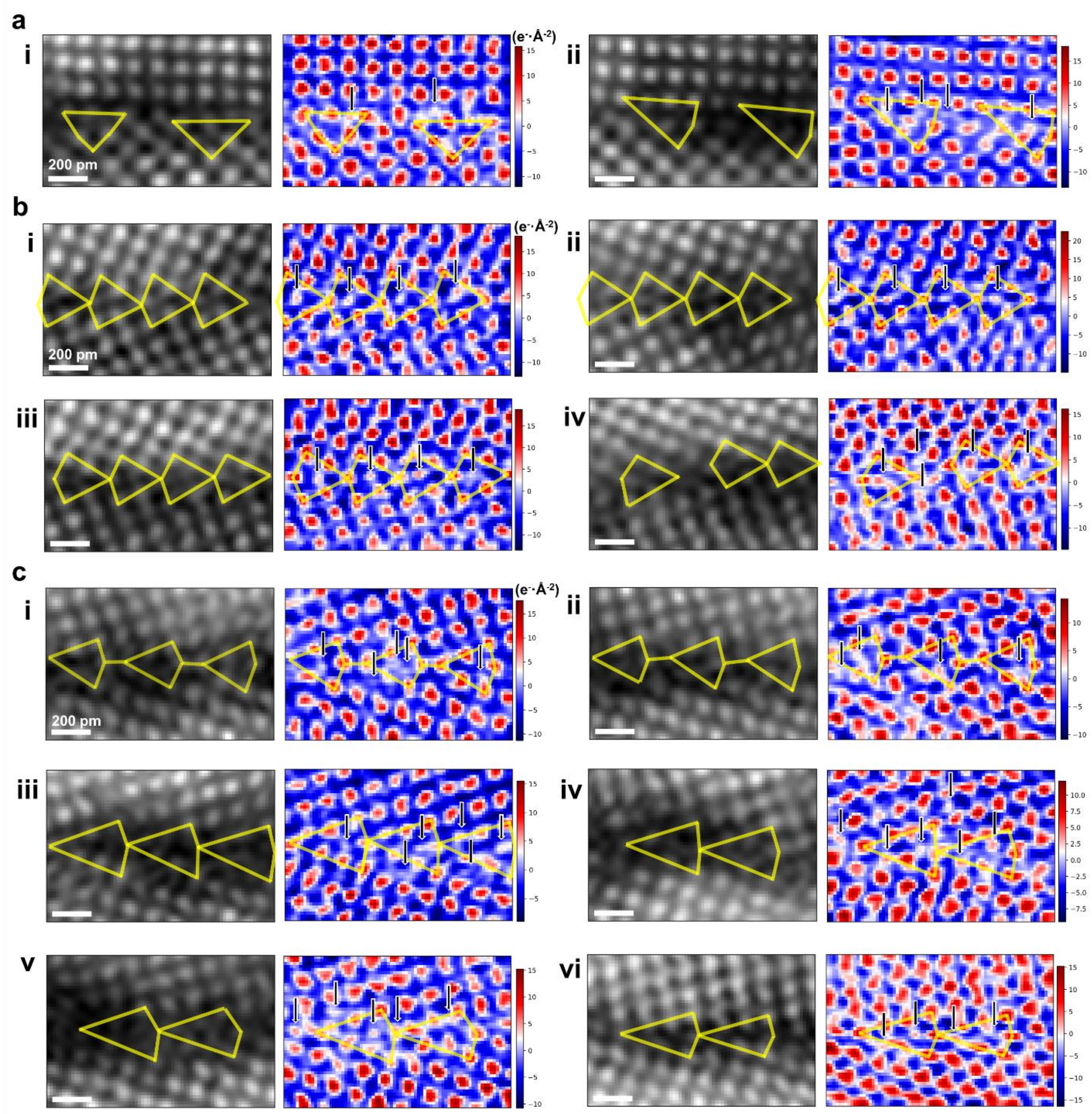

Supplementary Fig. 6 | Light solutes at Σ5 GBs for three representative atomic motifs: a i-ii (110) // (010); **b i-iv** (310) // (3$\bar{1}$0); **c i-vi** (2$\bar{1}$0) // (1$\bar{2}$0). For each atomic motif, the left and right images are the reconstructed dark-field and charge-density maps from the atomic DPC-4DSTEM data sets collected in different regions. The repeated structures at GBs have been highlighted in yellow lines. The atomic columns containing B or C are indicated by black arrows in the charge-density maps.

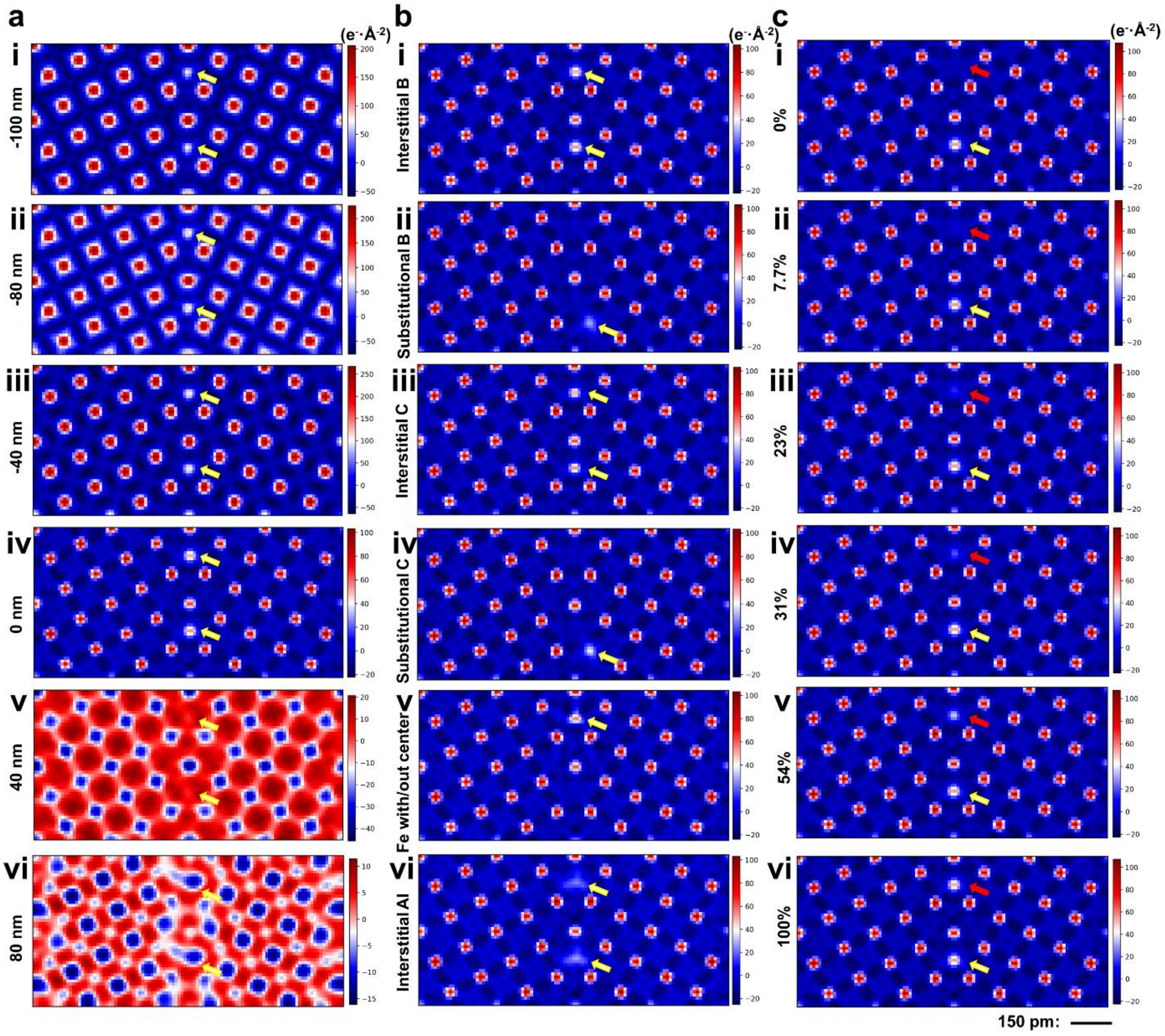

Supplementary Fig. 7 | The influence of defocus, solute type and structure, and site occupancy on the reconstructed charge-density maps from simulated DPC-4DSTEM data sets of the $\Sigma 5$ (310) // $(3\bar{1}0)$ atomic GB model. a i-vi Reconstructed charge-density maps with defocus from -100 nm to 80 nm. **b** Reconstructed charge-density maps for **i** interstitial B sites, **ii** substitutional B sites, **iii** interstitial C sites, **iv** substitutional C sites, **v** Fe sites in the kite center, and **vi** interstitial Al sites. **c i-vi** Reconstructed charge-density maps for the occupancy of B sites ranging from 0% to 100%, pointed by the red arrows. The other B atomic columns have 100% atom occupancy, pointed by the yellow arrows, serving as a reference.

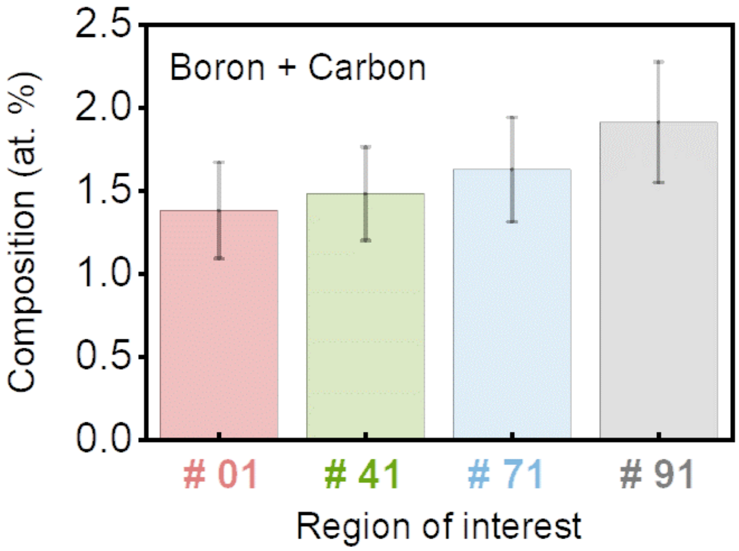

Supplementary Fig. 8 | Summary chart of local chemical compositions of Σ5 GBs for selected regions. The figure shows the average compositions of the sum of the boron and carbon content for the GBs of #01 (430) // (010), #41 (310) // ($3\bar{1}0$), #71 (11 1 0) // ($9\bar{8}0$), and #91 ($2\bar{1}0$) // ($1\bar{2}0$), respectively. The error bars indicate the standard deviations.

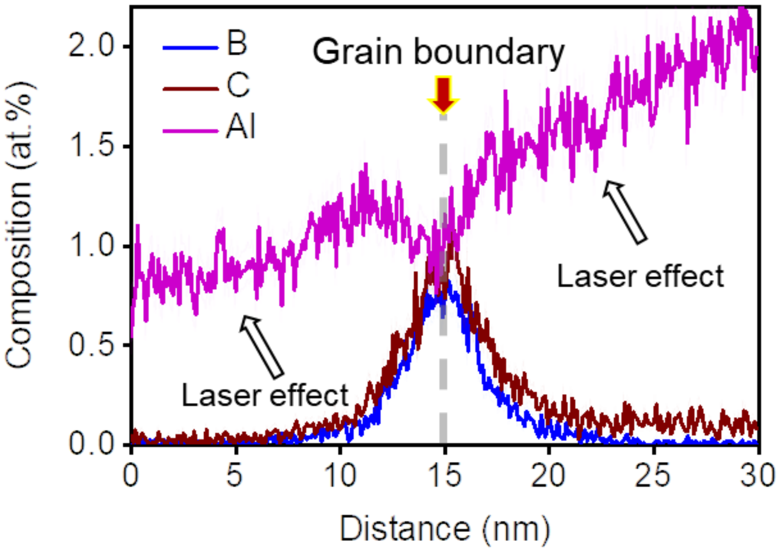

Supplementary Fig. 9 | One-dimensional (1D) line scan across the Σ5 (310)//(3$\bar{1}$0) GB. The enrichment of B and C and depletion of Al were observed in the GB region, indicated by the red arrow. The uneven distribution of Al, a gradient of Al content from the left to the right side, is due to the laser artifact [59]. The Al content is lower than its normal chemical composition because the Al^{+} peak overlaps with Fe^{2+}. Here we simply assigned the peak at 27Da (measured in Daltons, mass-to-charge ratio) to Fe^{2+} ions, resulting in a lower measurement of Al content, which is below its nominal value. A more detailed investigation can be found in our earlier publication [24]. The wide distribution observed in the segregation profiles of B and C, which can appear as thick as 10 nm, is primarily due to the local magnification artifact [60, 61]. Our DPC-4DSTEM results demonstrate that the interstitial atoms have primarily enriched the atomic motifs of the GBs, see Fig. 3 and Supplementary Fig. 6.

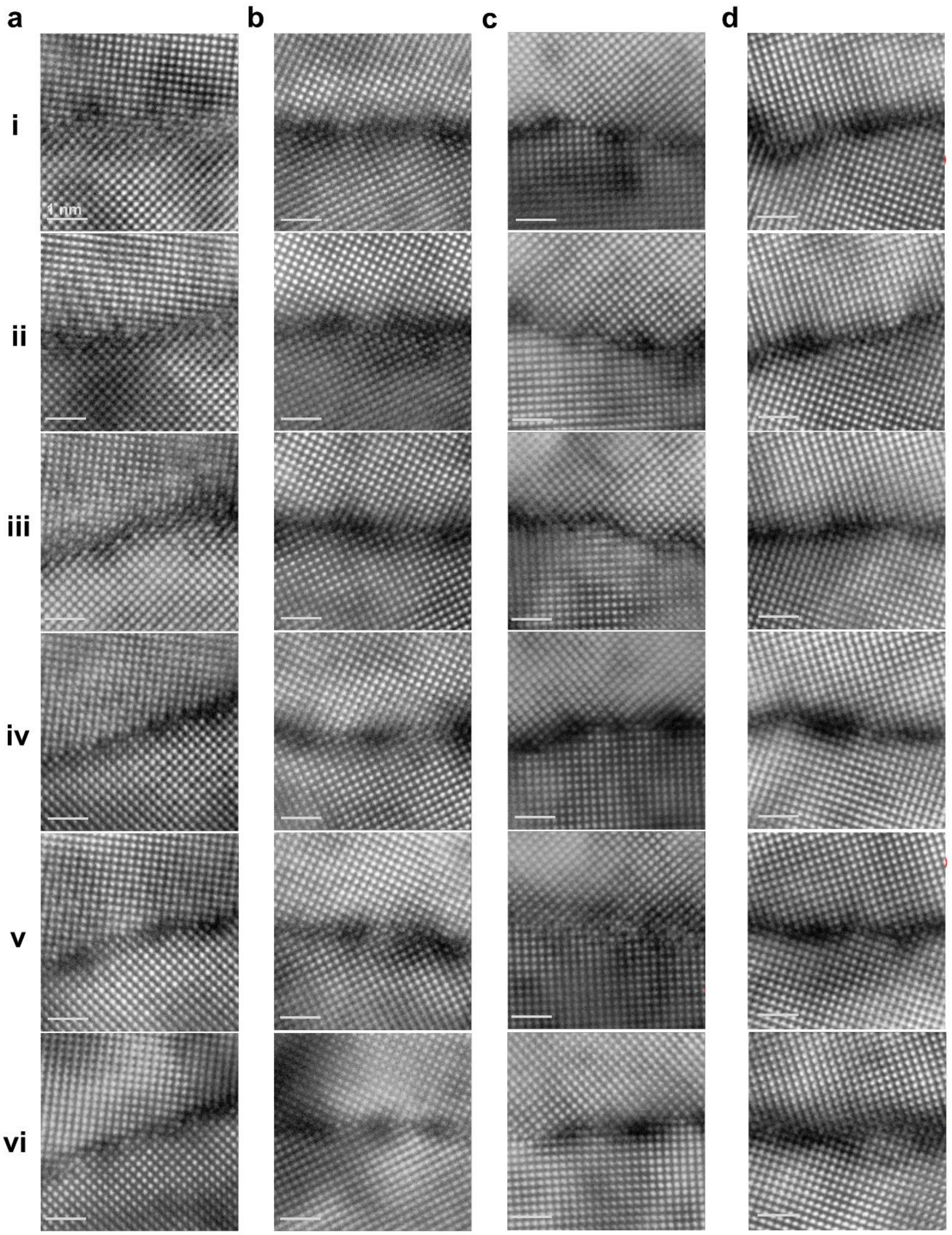

Supplementary Fig. 10 | Analysis of local facet structure of Σ5 GBs for selected regions. a-d are raw high resolution HAADF-STEM images of Σ5 GBs with mesoscopic boundary planes of #01 (430) // (010), #41 (310) // ($3\bar{1}0$), #71 (11 1 0) // ($9\bar{8}0$), and #91 ($2\bar{1}0$) // ($1\bar{2}0$), respectively.

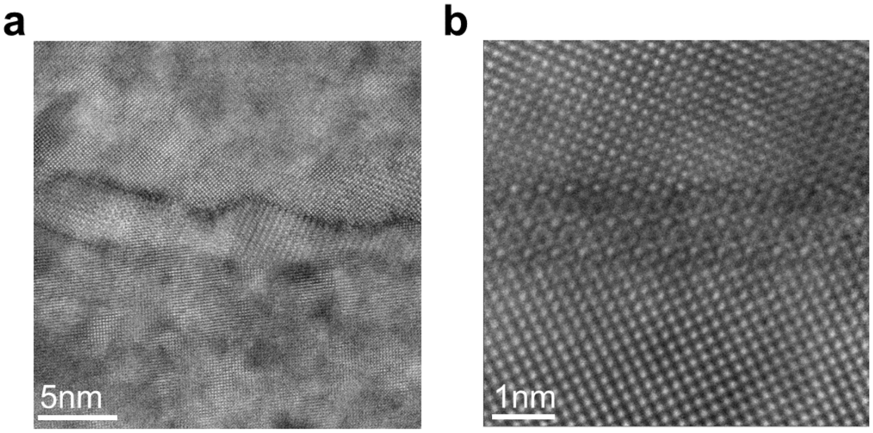

Supplementary Fig. 11 | HAADF-STEM images of selected Σ5 GBs to show areas where the top grain overlaps with the bottom grain in the direction perpendicular to the paper. a and **b** are Σ5 GBs with mesoscopic boundary planes of #01 (430) // (010) and #41 (310) // ($3\bar{1}0$), respectively. For these types of GBs, the local GB planes cannot be easily determined because they have both tilt and twist characters.

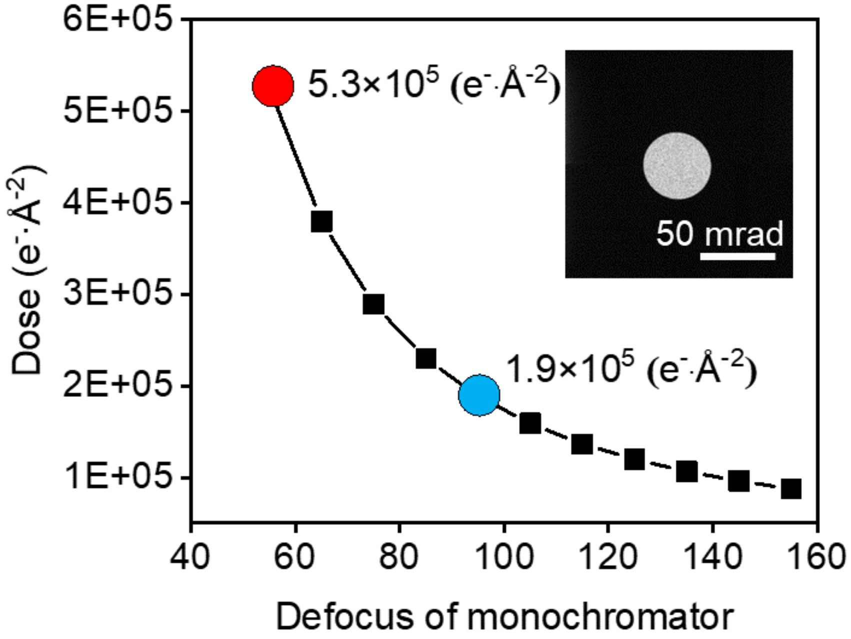

Supplementary Fig. 12 | Electron dose quantification with the Gatan camera for energy-filtered transmission electron microscopy (EFTEM). The red and blue dots show that the dose for 55 nA and 95 nA screen current is $5.3{\times}10^5$ $e^- \cdot Å^{-2}$ and $1.9{\times}10^5$ $e^- \cdot Å^{-2}$, respectively. These two values were used to acquire the atomic DPC-4DSTEM data sets. The inset gray image shows a reciprocal image of the probe hovering in the vacuum.

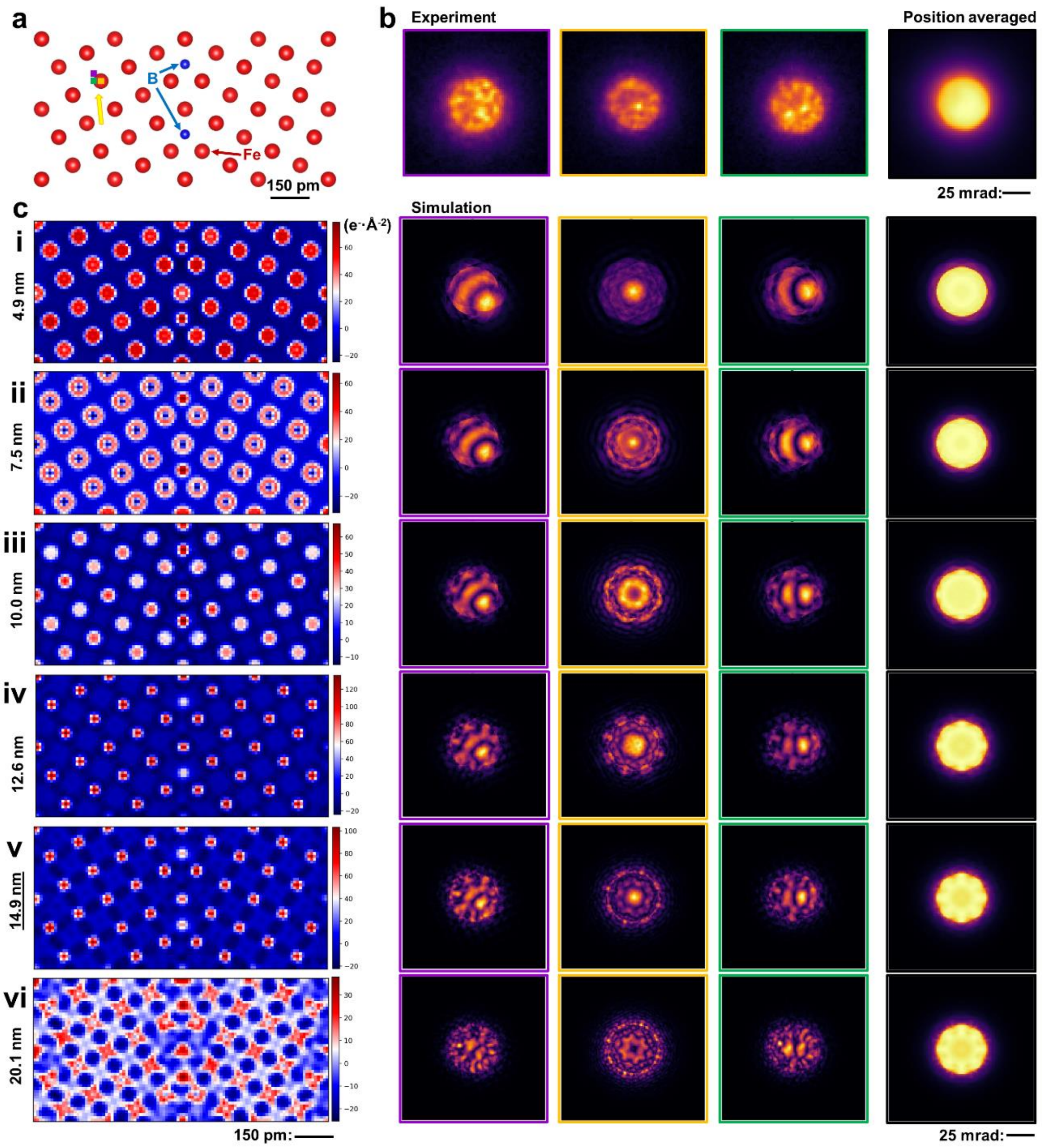

Supplementary Fig. 13 | Measurement of sample thickness by convergent beam electron diffraction (CBED). a The atomic model structure of the Σ5 Fe (colored red) GB with B (blue) decoration. **b** Experimental CBED patterns from the purple, orange, and green highlighted regions, respectively, of which positions are marked with a yellow arrow in a. Position-averaged CBED (PACBED) patterns were also shown in the rightmost column of each row. **c i-vi** Reconstructed charge-density maps (left image) and simulated CBED patterns (right three images) for the Σ5 (310) // ($3\bar{1}0$) GB model (see a) with thicknesses from 4.9 nm to 20.1 nm. Simulation results were calculated using μSTEM (v5.2) [57] and reconstructed using in-house developed Python scripts.

59. Sha G, Ringer SP. Effect of laser pulsing on the composition measurement of an Al–Mg–Si–Cu alloy using three-dimensional atom probe. *Ultramicroscopy* **109**, 580-584 (2009).

60. Miller M, Hetherington M. Local magnification effects in the atom probe. *Surf Sci* **246**, 442-449 (1991).

61. Vurpillot F, Bostel A, Blavette D. Trajectory overlaps and local magnification in three-dimensional atom probe. *Appl Phys Lett* **76**, 3127-3129 (2000).
